# Gender Disparities in Blood Pressure and the Role of Body Mass Index: A Birth Cohort Analysis in China

**DOI:** 10.1007/s44197-023-00127-y

**Published:** 2023-06-11

**Authors:** Jinjing Wu, Boshen Jiao, Jiaying Zhao

**Affiliations:** 1 Asian Demographic Research Institute, Shanghai University, Shanghai, 200444 China; 2 Harvard T.H. Chan School of Public Health, Harvard University, Boston, 02115 USA; 3 RSSS Building, 146 Ellery Crescent, School of Demography, ANU College of Arts and Social Sciences, The Australian National University, Acton ACT, Canberra, 2601 Australia

**Keywords:** Cardiovascular disease, Systolic/diastolic blood pressure, Sex/gender, Body mass index, China

## Abstract

**Background:**

The slow decline in cardiovascular disease (CVD) mortality and the stagnant or increasing hypertension prevalence in low- and middle-income countries necessitate investigation. Evolving gender disparities suggested that male cardiovascular health disadvantage may be preventable, offering potential for enhancing population cardiovascular health. Despite global body mass index (BMI) increases, its role in shaping the gender disparities remains underexplored.

**Objective:**

This study investigated the birth cohort dynamics of gender disparities in systolic/diastolic blood pressure (SBP/DBP) in China, one of the world's largest low- and middle-income countries, and explored the potential role of BMI in explaining the changing gender disparities.

**Methods:**

Data from the China Health and Nutrition Survey (1991–2015) were analyzed using multilevel growth-curve models to estimate gender- and cohort-specific SBP/DBP trajectories among individuals born between 1950 and 1975.

**Results:**

Men had higher SBP and DBP than women at the sample’s mean age of 41.7 years. The gender disparities in SBP and DBP increased with each successive one-year cohort from 1950 to 1975 by 0.14 mm Hg and 0.09 mm Hg, respectively. Adjusting for BMI reduced the increasing gender disparities in SBP and DBP by 31.9% and 34.4%, respectively.

**Conclusion:**

Chinese men experienced a greater increase in SBP/DBP across successive cohorts compared to women. The increasing gender disparities in SBP/DBP were partially attributable to a greater BMI increase across cohorts among men. Given these findings, prioritizing interventions that aim to reduce BMI, particularly among men, could potentially alleviate the burden of CVD in China through lowering SBP/DBP.

**Supplementary Information:**

The online version contains supplementary material available at 10.1007/s44197-023-00127-y.

## Introduction

Globally, cardiovascular disease (CVD) is the leading cause of premature death and a major contributor to disability for both genders [1]. The Global Burden of Disease (GBD) study showed a decline in the global age-standardized mortality rate of CVD between 1990 and 2019 [1], but high-income countries (HICs) experienced a greater decrease than low- and middle-income countries (LMICs) [2, 3]. The variations observed in the trends of age-standardized mortality rates of CVD between HICs and LMICs may be potentially attributed to disparities in CVD risk factor control [4]. Elevated blood pressure (BP) is a well-established risk factor for CVD morbidity and mortality, and its reduction is expected to significantly reduce the incidence of CVD [5, 6]. However, while the age-standardized hypertension prevalence decreased in HICs from 1990 to 2019, most LMICs experienced either an increase or stagnation [7], thereby making hypertension a growing concern in LMICs and shifting its burden from HICs to LMICs [8]. Therefore, it is crucial to identify and address local risk factors that may contribute to the stagnation of CVD mortality reduction and the rise or stagnation of hypertension prevalence in LMICs, in order to reduce the LMICs’ burden of CVD.

Existing research suggested that men generally exhibit higher systolic and diastolic blood pressure (SBP/DBP), and rates of CVD morbidity and mortality compared to women, at least until middle age [9]. However, the extent of gender disparities in these cardiovascular health indicators varies over time [9–12], indicating that the male disadvantage is not an inevitable outcome. By examining the dynamics of gender disparities in LMICs, we can identify gender-specific challenges and find opportunities to reduce the burden of CVD.

Research on the gender disparities in CVD morbidity in LMICs remains limited, although some studies have investigated recent trends in age-standardized CVD mortality rates by gender [13, 14]. However, declines in CVD mortality do not necessarily correspond to improvements in CVD morbidity if the CVD incidence remains constant or increases [15]. Furthermore, it’s worth noting that CVD is a condition that develops over a person’s lifetime, with current cardiovascular health reflecting the cumulative exposure to risk factors throughout life [16]. To gain a deeper understanding of gender-specific changes in CVD risk, it is essential to monitor the birth cohort dynamics of gender disparities in SBP/DBP and identify potential risk factors driving these disparities.

Being overweight or obese, defined as a body mass index (BMI) of 25 kg/m^2^ or higher, is a well-established risk factor for elevated BP and CVD morbidity [17]. Sedentary lifestyles and diets high in fat and animal products have resulted in a steady increase in global mean BMI from 1985 to 2017 [18]. The rise in BMI has been particularly pronounced in LMICs [18]. The increase in BMI in LMICs may exacerbate the male disadvantage in SBP/DBP due to gender stereotypes and interaction of sex-specific biological factors, including the hormone estrogen's cardioprotective effects [11, 16, 19, 20]. However, there is a lack of sufficient research in the existing literature investigating whether the rise in BMI contributes to the dynamics of gender disparities in SBP/DBP.

China is one of the largest LMICs, accounting for over one-sixth of the world's population. The country has undergone a rapid socioeconomic transformation in recent decades, transitioning from an agricultural-based economy to a primarily manufacturing-based economy. This transition has led to changes in dietary and physical activity patterns. Despite having lower average BMI and rates of overweight and obesity compared to Western societies, the Chinese population has experienced a concerning increase in BMI [21, 22]. An upward cohort trend in SBP/DBP observed in the Chinese population highlights the potential for a growing burden of CVD [23]. However, whether the cohort trend varies by gender and to what extent BMI can explain the gender heterogeneity in the cohort trend remain to be examined. Addressing these questions will provide insights into the epidemiological transition of CVD and strategies for mitigating the upward trend of SBP/DBP, ultimately reducing the burden of CVD.

This study utilized longitudinal data from the China Health and Nutrition Survey (CHNS, 1991–2015) to investigate two objectives. First, it aimed to examine whether men experienced a greater increase in SBP/DBP at the sample’s mean age compared to women for each successive one-year birth cohort from 1950 to 1975. Second, it aimed to explore the role of BMI in shaping the gender-specific cohort trend. China's experiences as a typical newly industrialized country may act as a sentinel for other LMICs undergoing similar socioeconomic transformations.

## Methods

### Data Source

We used longitudinal data from the China Health and Nutrition Survey (CHNS, 1991–2015), which collected valuable objective measures of SBP/DBP and anthropometric indicators and data on demographic, socioeconomic factors, lifestyles, and other health indicators. The survey covers twelve socioeconomically and geographically diverse provincial regions, including Heilongjiang, Liaoning, Shandong, Henan, Jiangsu, Hubei, Hunan, Guizhou, Guangxi, Beijing, Shanghai, and Chongqing. Figure S1 in Supplementary Materials illustrates the geographical distribution of these survey regions.

The CHNS survey utilized a multistage, random cluster sampling to draw households and surveyed all household members. Although the CHNS is not nationally representative, the CHNS sample’s characteristics are comparable to those from nationally representative samples [24, 25].

The CHNS spanned more than 20 years, from 1989 to 2015. It undertook the first survey in 1989, with follow-up surveys in 1991, 1997, 2000, 2004, 2006, 2009, 2011, and 2015, respectively. We did not use data collected in 1989 since this wave only included people aged 20–45. It was difficult to determine the CHNS’s response rate because respondents who were lost to follow-up in one survey could come back in a later survey [25]. Furthermore, the CHNS recruited new participants as replenishment samples since 1997 [25].

We presented the sample selection process in Figure S2 in Supplementary Materials. Our analyses were limited to people who (1) were born during 1950–1975 and (2) were not pregnant during the survey. A total of 16,318 respondents, with 70,318 observations, were eligible for this study. We conducted complete-case analyses by excluding 1,943 respondents (11.9% of 16,318 respondents) with missing values for SBP/DBP, BMI, and control variables. Our final analysis included 14,375 respondents, with 49,467 observations. The average follow-up time for each respondent was about 8.7 years. Of 14,375 respondents, 37.3% were surveyed in other waves but were censored in the 2015 wave. There were 2.4% of respondents dying by the 2015 wave. Methods for dealing with potential bias resulting from missing values, loss to follow-up, and deaths are introduced below.

### Variables

#### Dependent Variables

We used objectively measured SBP/DBP as a proxy for CVD. In each wave, trained healthcare professionals measured each respondent's SBP/DBP three times at one-minute intervals using a mercury sphygmomanometer after respondents rested for at least five minutes. We used the average of the three SBP/DBP measurements as our dependent variables. We preferred continuous variables of SBP/DBP over a binary variable of hypertension, as prior evidence suggested that SBP/DBP was positively associated with CVD morbidity and mortality, even within normal range [5, 26].

#### Independent Variables

Independent variables in this study included gender, birth cohort, and BMI. We created a continuous cohort variable by centering the individuals’ birth years at 1950, the sample’s earliest birth year. Trained healthcare professionals assessed weight (accurate to 0.1 kg while wearing light clothing) using a balance-beam scale, and height (accurate to 0.1 cm without shoes) using a stadiometer in each survey. BMI was calculated as weight (in kg) divided by the square of height (in meters). The calculated BMI values were then centered at the sample’s mean BMI.

#### Control Variables

We included demographic variables (age, marital status), socioeconomic variables (educational attainment, household income per capita, employment, urban/rural residence), and risky behaviors (smoking, frequent alcohol drinking) as covariates in our analysis. We centered the age variable at the sample’s mean age of 41.7 years. We classified educational attainment into four categories: primary education or below (reference group), lower secondary education, upper secondary education, and tertiary education or above. Household income per capita was adjusted for the 2015 Consumer Price Index. Marital status (0 = not married, 1 = married), employment (0 = unemployed, 1 = employed), place of residence (0 = rural residence, 1 = urban residence), smoking (0 = never smoked, 1 = ever smoked), and alcohol drinking (0 = not a frequent alcohol drinker, 1 = frequent alcohol drinker) were coded as dummy variables.

We also included provincial fixed effects to account for substantial regional variations. Furthermore, we included time-constant dummy variables indicating the loss to follow-up and the deceased to account for the potential bias resulting from loss to follow-up and deaths. Table S1 in Supplementary Materials reports the measurement and classification of all variables included in this study.

### Statistical Analyses

In descriptive analyses, we employed the chi-square test and the t-test to investigate potential gender disparities in categorical and continuous variables, respectively. To extend our research, we used multilevel growth-curve models to assess the longitudinal SBP/DBP trajectories. We added age and age squared terms as previous studies have reported a nonlinear relationship between age and SBP/DBP [23]. We further included cohort, the interaction between cohort and age, and the interaction between cohort and age squared to test the cohort variations in the SBP/DBP trajectories. We did not account for the period effect as the period variable perfectly overlapped with the age variable in longitudinal studies. To examine the gender disparities in the SBP/DBP trajectories, we included gender, the interaction between gender and age, and the interaction between gender and age squared (Model 1). To examine whether the gender disparities in SBP/DBP at the sample’s mean age changed across successive cohorts, we included an interaction between gender and cohort (Model 2). Furthermore, we assessed the role of BMI in the changing gender disparities across successive cohorts by comparing the estimates of the interaction between gender and cohort before and after including the BMI variable (Model 3).

$${\mathrm{y}}_{\mathrm{ti}}={\upbeta }_{0}+{\upbeta }_{1}\mathrm{AGE}+{\upbeta }_{2}{\mathrm{AGE}}^{2}+{\upbeta }_{3}\mathrm{GENDER}+{\upbeta }_{4}\mathrm{ GENDER}\bullet \mathrm{AGE}+ {\upbeta }_{5}\mathrm{ GENDER }\cdot {\mathrm{AGE}}^{2}$$

$${+\upbeta }_{6}\mathrm{COHORT}+{\upbeta }_{7}\mathrm{ COHORT}\bullet \mathrm{AGE}+{\upbeta }_{8}\mathrm{ COHORT}\bullet {\mathrm{AGE}}^{2}+$$

$${+\upbeta }_{9}\mathrm{GENDER}\bullet \mathrm{COHORT}+{\upbeta }_{10}\mathrm{BMI}+{\upbeta }_{11}\mathrm{Z}+{\upmu }_{0\mathrm{i}}+{\mathrm{e}}_{\mathrm{ti}}$$

All variables included in the models are time-varying, with the exception of gender, cohort, provincial regions, loss to follow-up, and deaths. We used the "mixed" commands in STATA 17.0 to perform the analyses. All tests conducted in this study utilized a two-tailed test, with the significance level ($\mathrm{\alpha }$) set at 0.05.

### Sensitivity Analyses

We conducted three sensitivity analyses to assess the robustness of our findings. In the first sensitivity analysis, we evaluated whether our primary findings remained when using hypertension as a dependent variable. Based on the 2018 European Society of Cardiology/European Society of Hypertension Guidelines, respondents were classified as hypertensive if their SBP/DBP levels were equal to or greater than 140/90 mm Hg [27]. Additionally, we categorized respondents as hypertensive if they either had a diagnosis of hypertension or were taking antihypertensive medication. We employed the “melogit” command in Stata 17 to construct the multilevel logistic model.

In the second sensitivity analysis, we examined whether antihypertensive treatment influenced our findings by excluding individuals who were taking antihypertensive drugs from our analyses.

In the third sensitivity analysis, we examined whether our findings were robust to missing data by including all eligible respondents in our analyses. We took three steps to fill in the missing values of the dependent, independent, and control variables. First, we used the “mi chained” command in Stata 17 to generate ten multiply imputed datasets. Second, we rebuilt the multilevel linear model for each multiply imputed dataset. Third, we pooled regression coefficients and their standard errors across the ten multiply imputed datasets.

## Results

Based on each respondent's baseline observation, Table 1 reports descriptive statistics of the dependent, independent, and control variables by gender.

**Table 1:** Descriptive statistics of dependent, independent, and control variables by sex at baseline, the China Health and Nutrition Survey (1991–2015)

Continuous variables		Males	Females	*P-*Value
		Mean (SD)	Mean (SD)	
Systolic blood pressure level, mm Hg		118.38 (0.18)	113.16 (0.18)	< 0.001
Diastolic blood pressure level, mm Hg		77.63 (0.13)	74.08 (0.12)	< 0.001
Baseline age (year)		36.67 (0.14)	36.34 (0.13)	0.05
Household income per capita, in thousands of Chinese yuan		8.93 (0.17)	8.98 (0.21)	0.42

Categorical variables		n (%)	n (%)	*P-*Value
Marital status	Married	5,417 (79.64)	6,298 (83.16)	< 0.001
Place of residence	Urban	2,746 (40.37)	3,098 (40.91)	0.26
Education	Primary education or below (reference group)	1,679 (24.68)	2,684 (35.44)	< 0.001
	Lower secondary education	2,819 (41.44)	2,788 (36.82)	< 0.001
	Upper secondary education	1,331 (19.57)	1,240 (16.37)	< 0.001
	Tertiary education	973 (14.30)	861 (11.37)	< 0.001
Employment	Employed	5,781 (84.99)	5,575 (73.62)	< 0.001
Smoking	Ever smoked	4,368 (64.22)	169 (2.23)	< 0.001
Alcohol drinking	Frequent alcohol drinker	1,769 (26.01)	104 (1.37)	< 0.001
Follow-up status	Loss to follow-up	2,557 (37.59)	2,802 (37.08)	0.26
Survival status	Dead	209 (1.72)	130 (3.07)	< 0.001
Number of respondents		6,802	7,573	–

*SD* Standard deviation

Table 2 reports the estimates from the multilevel growth-curve models of both SBP and DBP. In Model 1 of SBP/DBP, we included gender, cohort, and all control variables. The coefficient of gender indicated that men had higher SBP/DBP at the sample's mean age than women. The coefficient of the cohort implied that SBP/DBP at the sample's mean age increased for each successive one-year birth cohort from 1950 to 1975.

**Table 2:** Estimates from multilevel growth-curve models of systolic blood pressure (SBP) and diastolic blood pressure (DBP), China Health and Nutrition Survey (1991–2015)

	SBP			DBP		
	Model 1	Model 2	Model 3	Model 1	Model 2	Model 3
	Coefficient	Coefficient	Coefficient	Coefficient	Coefficient	Coefficient
	(95% CI)	(95% CI)	(95% CI)	(95% CI)	(95% CI)	(95% CI)
Fixed effects						
Intercept	111.92 (110.63–113.20)	112.71 (111.38–114.04)	112.11 (110.83–113.40)	72.80 (71.92–73.68)	73.32 (72.41–74.23)	72.89 (72.02–73.77)
Male (Reference = Female)	4.73 (4.23–5.24)	2.98 (2.09–3.87)	3.26 (2.41–4.12)	3.74 (3.39–4.09)	2.59 (1.98–3.20)	2.79 (2.20–3.37)
Birth cohort	0.38 (0.35–0.41)	0.31 (0.27–0.36)	0.26 (0.22–0.31)	0.22 (0.20–0.24)	0.18 (0.15–0.21)	0.14 (0.11–0.17)
Male*Birth cohort	–	0.14 (0.08–0.20)	0.09 (0.04–0.15)	–	0.09 (0.05–0.13)	0.06 (0.02–0.10)
Body mass index (kg/m^2^)	–	–	0.92 (0.87–0.96)	–	–	0.65 (0.62–0.68)
Control variables						
Age	0.78 (0.73–0.83)	0.76 (0.71–0.81)	0.66 (0.61–0.71)	0.47 (0.44–0.50)	0.46 (0.42–0.49)	0.38 (0.35–0.42)
Age squared	0.01 (0.01–0.02)	0.01 (0.01–0.02)	0.01 (0.01–0.02)	− 0.00 (− 0.01–0.00)	− 0.00 (− 0.01–0.00)	− 0.00 (− 0.01–0.00)
Male*Age	− 0.14 (− 0.17–0.11)	− 0.10 (− 0.14–0.07)	− 0.11 (− 0.14–0.07)	− 0.03 (− 0.05–0.01)	− 0.00 (− 0.03–0.02)	− 0.01 (− 0.03–0.01)
Male*Age squared	− 0.00 (− 0.01–0.00)	− 0.00 (− 0.01–0.00)	− 0.00 (− 0.00–0.00)	− 0.00 (− 0.00–0.00)	− 0.00 (− 0.00–0.00)	− 0.00 (− 0.00–0.00)
Birth cohort*Age	0.01 (0.01–0.02)	0.01 (0.01–0.02)	0.01 (0.01–0.01)	0.00 (0.00–0.01)	0.00 (0.00–0.01)	0.00 (0.00–0.01)
Birth cohort*Age squared	0.00 (0.00–0.00)	0.00 (0.00–0.00)	0.00 (0.00–0.00)	0.00 (0.00–0.00)	0.00 (0.00–0.00)	0.00 (0.00–0.00)
Married (Reference = Not married)	− 0.31 (− 0.83–0.21)	− 0.31 (− 0.83–0.21)	− 0.43 (− 0.94–0.08)	− 0.24 (− 0.60–0.12)	− 0.24 (− 0.60–0.11)	− 0.33 (− 0.68–0.02)
Urban (Reference = Rural)	− 0.61 (− 1.01–0.21)	− 0.61 (− 1.01–0.21)	− 0.71 (− 1.09–0.33)	− 0.31 (− 0.58–0.04)	− 0.31(− 0.58–0.04)	− 0.38 (− 0.63–0.12)
Education (Reference = Primary education or below)	1	1	1	1	1	1
Lower secondary education	− 0.41 (− 0.79–0.04)	− 0.38 (− 0.76–0.01)	− 0.41 (− 0.78–0.05)	− 0.05 (− 0.31–0.21)	− 0.03 (− 0.29–0.23)	− 0.05 (− 0.30–0.20)
Upper secondary education	− 0.55 (− 1.04–0.06)	− 0.53 (− 1.02–0.04)	− 0.51 (− 0.99–0.04)	− 0.34 (− 0.68–0.01)	− 0.33 (− 0.67–0.01)	− 0.29 (− 0.62–0.03)
Post-secondary education	− 1.79 (− 2.39–1.20)	− 1.78 (− 2.37–1.18)	− 1.67 (− 2.24–1.09)	− 0.89 (− 1.30–0.49)	− 0.88 (− 1.29–0.47)	− 0.77 (− 1.16–0.38)
Household income per capita (in thousands of Chinese yuan)	− 0.00 (− 0.01–0.00)	− 0.00 (− 0.01–0.00)	− 0.00 (− 0.01–0.00)	0.00 (− 0.00–0.01)	0.00 (− 0.00–0.01)	0.00 (− 0.00–0.00)
Employed (Reference = Not employed)	0.11 (− 0.21–0.42)	0.10 (− 0.22–0.41)	0.18 (− 0.13–0.49)	0.11 (− 0.11–0.33)	0.11 (− 0.12–0.33)	0.15 (− 0.07–0.37)
Ever smoking (Reference = Never smoking)	0.12 (− 0.27–0.51)	0.15 (− 0.24–0.54)	0.39 (0.00–0.77)	− 0.01 (− 0.28–0.26)	0.01 (− 0.26–0.29)	0.18 (− 0.08–0.45)
Frequent alcohol drinking (Reference = No frequent alcohol drinking)	1.00 (0.61–1.39)	1.04 (0.65–1.43)	1.03 (0.65–1.42)	0.57 (0.29–0.84)	0.59 (0.32–0.87)	0.60 (0.33–0.87)
Loss to follow-up	− 0.16 (− 0.58–0.26)	− 0.17 (− 0.58–0.25)	− 0.16 (− 0.56–0.24)	0.20 (− 0.08–0.48)	0.19 (− 0.09–0.48)	0.19 (− 0.07–0.46)
Death	3.02 (1.79–4.25)	3.11 (1.87–4.34)	3.43 (2.25–4.60)	1.11 (0.28–1.94)	1.16 (0.33–2.00)	1.41 (0.62–2.19)
Provincial fixed effect	Yes	Yes	Yes	Yes	Yes	Yes
Random-effect variance components						
Individual-level: in intercept	63.23	63.03	52.79	27.92	27.84	22.88
Individual-level: in slope	0.13	0.13	0.12	0.03	0.03	0.03
Residual	126.11	126.08	125.62	65.24	65.23	65.04
AIC	395,707.49	395,687.76	394,088.07	360,471.92	360,453.74	358,776.94
Number of respondents (Number of observations)	14,375 (49,467)	14,375 (49,467)	14,375 (49,467)	14,375 (49,467)	14,375 (49,467)	14,375 (49,467)

*95% CI* Confidence interval, *AIC* Akaike information criterion

**Fig. 1:**
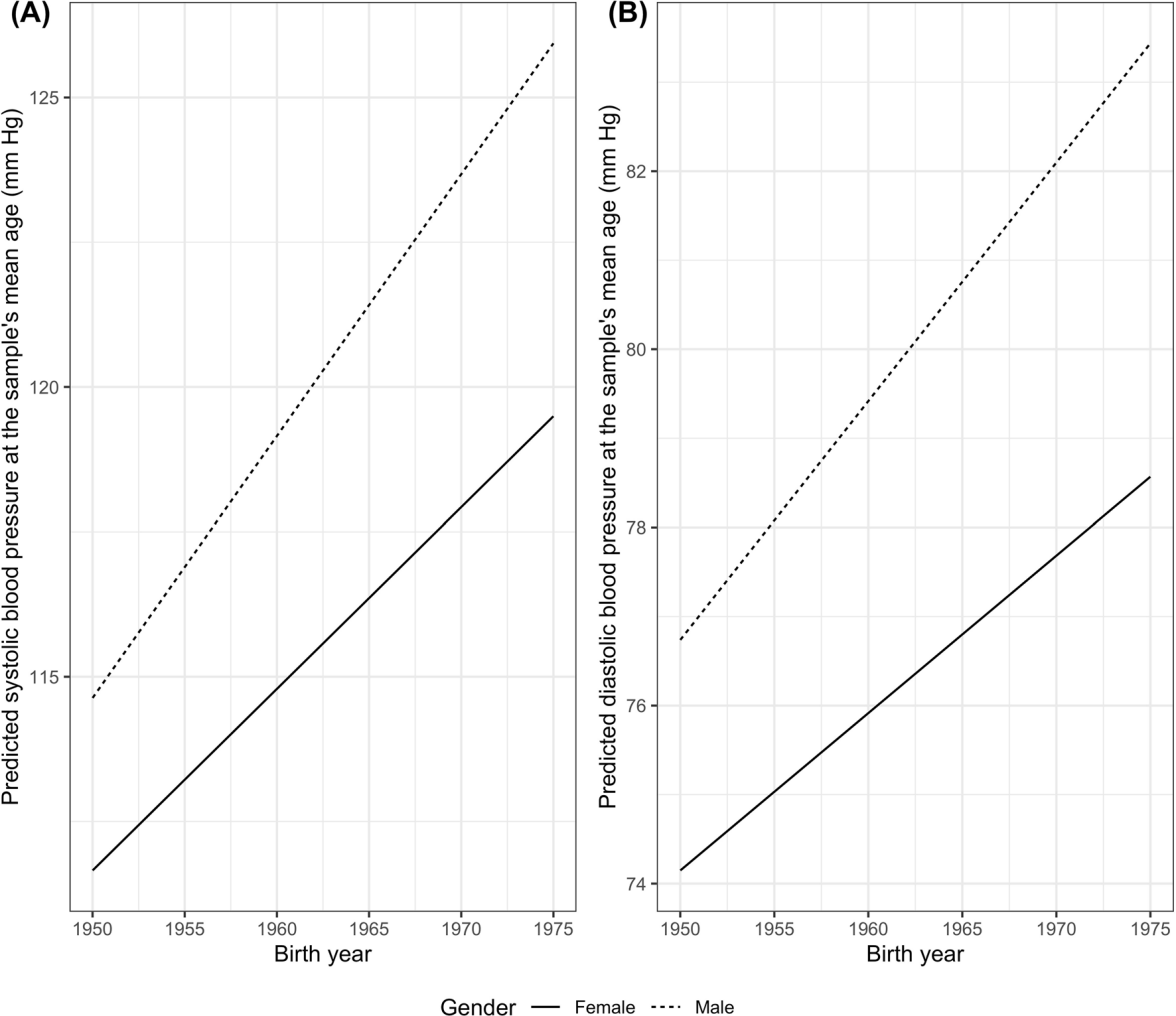
Estimated systolic blood pressure (SBP) (**A**) and diastolic blood pressure (DBP) (**B**) at the sample's mean age (41.7 years), China Health and Nutrition Survey (1991–2015)

In Model 2 of SBP/DBP, we further included the interaction between gender and cohort. The interaction coefficient indicated that men had a greater increase in SBP/DBP at the sample's mean age across successive cohorts than women. Figure 1 shows that the gender disparities in either SBP or DBP at the sample's mean age increased across successive cohorts. The gender disparities in SBP and DBP increased by 0.14 mm Hg (95% confidence interval [CI]: 0.08, 0.20) and 0.09 mm Hg (95% CI: 0.05, 0.13), respectively, for each successive one-year birth cohort from 1950 to 1975.

**Fig. 2:**
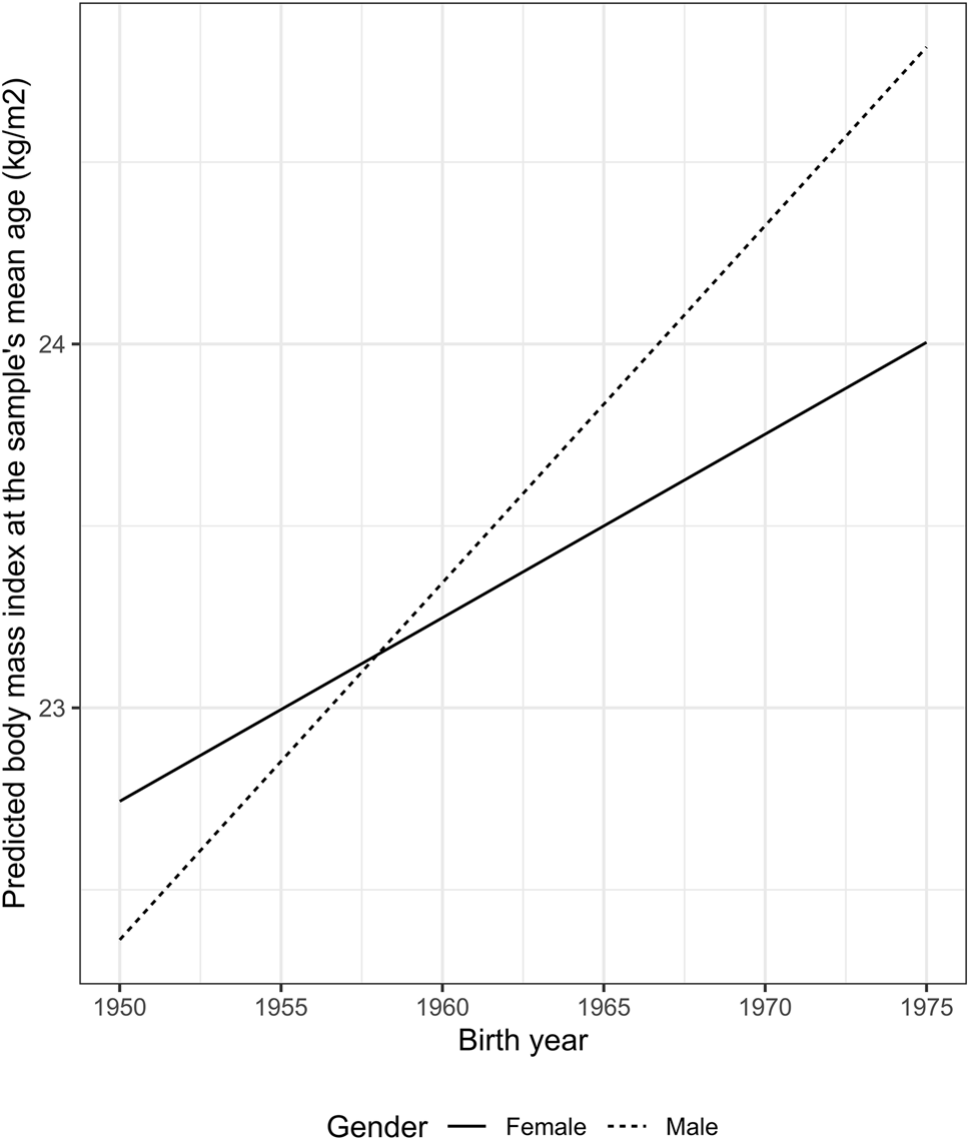
Estimated body mass index (BMI) at the sample's mean age (41.7 years), China Health and Nutrition Survey (1991–2015)

Before assessing the impact of BMI on the gender disparities in SBP/DBP, we first examined the evolving gender disparities in BMI across successive cohorts. Table S2 in the Supplementary Materials presents the estimates of the cohort trend. The coefficient of gender in the BMI model indicated that, on average, men had a lower BMI level than women at the sample’s mean age. Furthermore, the coefficient of the interaction between gender and cohort indicated that men experienced a greater increase in BMI at the sample’s mean age across successive cohorts compared to women. Notably, while women had a higher BMI level at the sample’s mean age than men in older cohorts, the gender disparities reversed and expanded across successive cohorts (Fig. 2).

To assess the impact of BMI on the cohort trend of the gender disparities in SBP/DBP, we extended Model 2 by including BMI in Model 3. Our analysis showed that adjusting for BMI resulted in a reduction of 31.9% and 34.4% in the increasing gender disparities in SBP and DBP across successive cohorts, respectively.

The Supplementary Materials report the estimates from our sensitivity analyses. Our findings remained consistent even after using hypertension as the dependent variable (Table S3), excluding individuals who received antihypertensive treatment (Table S4), or using the multiply imputed datasets (Table S5).

## Discussion

The principal finding of this study was that Chinese men had a disproportionate increase in SBP/DBP at the sample’s mean age for each successive one-year cohort compared with Chinese women, resulting in an expansion of the gender disparities in SBP/DBP across successive cohorts. Additionally, men had a greater increase in BMI at the sample’s mean age across successive cohorts than women. After adjusting for BMI, the upward cohort trend of gender disparities in SBP/DBP decreased by over 30%, suggesting that BMI can partly explain the increasing gender disparities.

A prior study compared cohort trends of male-to-female ratios of CVD mortality in several East Asian countries or regions (i.e., Japan, South Korea, Hong Kong, and Taiwan) and Western countries (i.e., Australia, England and Wales, Sweden, and France) [19]. It reported that for people born post 1946–1950, the male-to-female CVD mortality ratios increased across successive cohorts in East Asia, while these ratios either decreased or stabilized in Western countries [19]. However, the focus of this study was predominantly on gender disparities in CVD mortality, and its analyses were confined to high-income countries or regions. Our research extends the previous study by revealing an upward cohort trend in gender disparities in SBP/DBP in China. This upward cohort trend aligns with the increasing male-to-female ratios of CVD mortality across successive cohorts observed in East Asian regions [19].

The discrepancy in findings between Western and East Asian regions, including China, may be attributed to variations in smoking trends. In HICs, men had a more significant decrease in smoking rates than women, leading to a narrowing gender gap in smoking prevalence [28]. The decreasing gender gap in smoking has been identified as a significant contributor to reducing gender disparities in all-cause mortality and CVD mortality, although the extent to which smoking explains the gender disparities varies across countries [29]. However, data from China suggested that while female smoking prevalence remained low and showed a decrease across successive cohorts, male smoking prevalence remained high, with no substantial decrease across cohorts [30]. Examining the role of smoking in the increasing gender disparities in SBP/DBP goes beyond this paper's scope and necessitates future research.

One prior cross-country ecological study found a positive correlation between fat intake and the male-to-female ratio of mortality from coronary heart disease, a major type of CVD [10]. Our study built upon the prior study by providing insights into the role of BMI in shaping gender disparities in SBP/DBP at the individual level. Specifically, our findings, which showed a greater increase in BMI at the sample’s mean age across successive cohorts among men compared to women, align with the findings of the prior ecological study [10].

Our findings were consistent with a previous study that found a faster increase in BMI and obesity in cohort succession in men than in women in China [22]. These uneven cohort trends of BMI between genders may be explained by gender norms, where women are stereotypically expected to eat less and prioritize health, while men are often expected to consume larger portions and higher-fat diets [31]. Additionally, cultural ideals of femininity often prioritize slenderness [32], which may lead to higher body dissatisfaction and weight-control efforts among women than men [33]. These factors could explain the greater increase in BMI among men than women across successive cohorts.

Conversely, research conducted in the US suggested that women had a stronger upward cohort trend of obesity compared to men [34]. These inconsistent findings between China and Western countries, along with the evidence on smoking [28, 30], suggested that cultural factors may shape gender disparities in lifestyles and, consequently, cardiovascular health outcomes. In Western countries, where gender roles are evolving towards greater equality, women are increasingly adopting behaviors that have traditionally been associated with men, such as smoking and consuming high-fat foods [12]. However, in East Asian societies, Confucian Patriarchal norms and gender traditional role differentiation continue to exert a significant influence [35, 36]. While adherence to feminine roles may discourage women from adopting risky behaviors, gender discrimination may expose women to mental health issues that detrimentally affect their cardiovascular health outcomes [37]. Further research is warranted to enhance our understanding of the influence of cultural factors and gender norms on cohort trends of gender disparities in SBP/DBP.

While this study provides valuable insights, it is subject to several limitations. Firstly, although the CHNS includes a diverse range of economically and geographically representative regions across China, it is not nationally representative. Therefore, the findings of this study cannot be generalized to the entire country but only to the twelve provincial regions included in the study. Secondly, the study used SBP/DBP as a proxy for assessing CVD risk, as the role of elevated blood pressure in causing CVD morbidity and mortality has been well-established through high-quality observational studies and randomized controlled trials [5, 6]. However, further studies should consider other CVD risk factors and the incidence and prevalence of CVD to comprehensively understand the epidemiological transition of CVD and its gender heterogeneity. Thirdly, we excluded people born after 1975 from our analyses due to the unavailability of SBP/DBP data in their middle age. Once more data become available from younger cohorts, the studies should be updated to include these cohorts.

## Conclusion

Our research offered new insights into the epidemiological transition of CVD and its gender heterogeneity by investigating the cohort dynamics of gender disparities in SBP/DBP—a significant contributor to CVD morbidity and mortality—in China. The gender-specific cohort trend of SBP/DBP was partially attributed to a more pronounced increase in BMI across successive cohorts among men than women. These findings have important public health implications. Targeting interventions to reduce increasing BMI, particularly among men, could potentially alleviate the burden of CVD in China through lowering SBP/DBP.

## Supplementary Information

Below is the link to the electronic supplementary material.

Supplementary file1 (DOCX 219 KB)

## Data Availability

The datasets generated during and/ or analyzed during the current study are available in the China Health and Nutrition Survey repository, [https://www.cpc.unc.edu/projects/china].

## Abbreviations

BP: Blood pressure
SBP: Systolic blood pressure
DBP: Diastolic blood pressure
BMI: Body mass index
CVD: Cardiovascular disease
CHNS: China Health and Nutrition Survey
HICs: High-income countries
LMICs: Low- and middle-income countries
