# Supplementary material for: Gender Disparities in Blood Pressure and the Role of Body Mass Index: A Birth Cohort Analysis in China
Source: J Epidemiol Glob Health. 2023 Jun 11;13(3):485–94. doi: 10.1007/s44197-023-00127-y (PMC10468457; doi:10.1007/s44197-023-00127-y)
Supplement: Supplementary file 1 — Supplementary file1 (DOCX 219 KB) [file 44197_2023_127_MOESM1_ESM.docx]

***Journal of Epidemiology and Global Health***

**Supplementary Materials**

**Gender Disparities in Blood Pressure and the Role of Body Mass Index: A Birth Cohort Analysis in China**

**Jinjing Wu^1^, Boshen Jiao^2^, Jiaying Zhao^3*^**

^1^ Asian Demographic Research Institute, Shanghai University, 200444 Shanghai, China.

^2^ Harvard T.H. Chan School of Public Health, Harvard University, 02115 Boston, USA.

^3^ College of Arts and Social Sciences, Australian National University, 2601 Canberra, Australia.

***Corresponding author** Jiaying Zhao, Ph.D., RSSS Building, 146 Ellery Crescent, School of Demography, ANU College of Arts and Social Sciences, The Australian National University, Acton ACT 2601, Australia. Email: zjl789@gmail.com; Tel: 61-423966870.


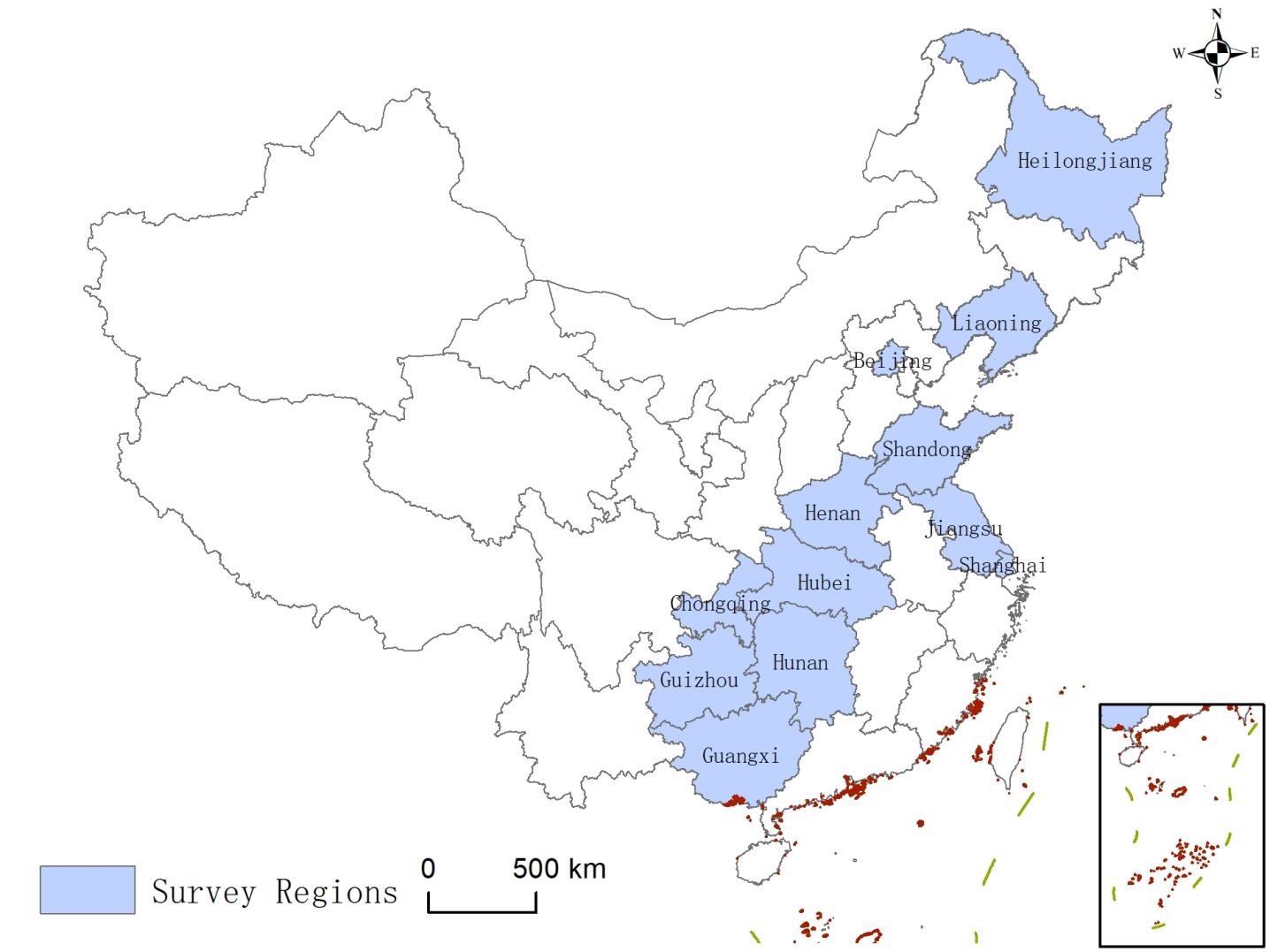


**Figure S1** The survey regions of the China Health and Nutrition Survey (1991-2015)


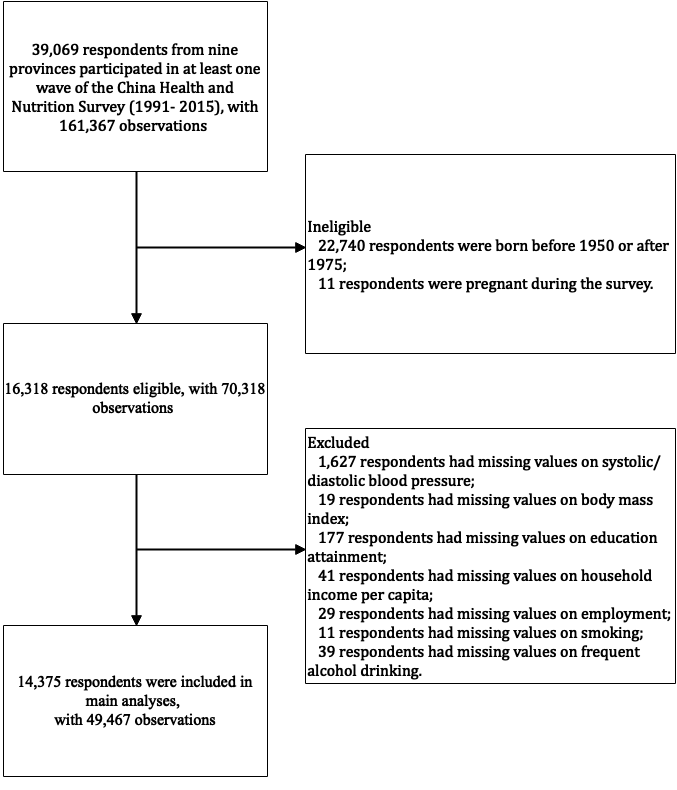


**Figure S2** Sample selection process, China Health and Nutrition Survey (1991-2015)

**Table S1** The measurements and classifications of dependent, independent, and control variables, the China Health and Nutrition Survey (1991-2015)

| **Dependent variables** | **Measurement questions** | **Original response categories** | **Analytical categories** |
| --- | --- | --- | --- |
| Systolic blood pressure level (mm Hg) | The assessment of blood pressure was conducted by trained healthcare professionals including physicians, nurses, and other healthcare workers | Systolic blood pressure levels: _mm Hg | Continuous variable |
| Diastolic blood pressure level (mm Hg) |  | Diastolic blood pressure levels: _mm Hg | Continuous variable |
| **Independent variables** |  |  |  |
| Male | Sex: | 1=Male 2=Female | 1=Male 0=Female |
| Birth cohort | Date of birth:_year_month_day | Birth year | Continuous variable |
| Body mass index (kg/m^2^) | The assessment of weight and height was conducted by trained healthcare professionals including physicians, nurses, and other healthcare workers. Trained healthcare professionals assessed weight (accurate to 0.1 kg while wearing light clothing) using a balance-beam scale, and height (accurate to 0.1 cm without shoes) uses a stadiometer. | Weight: _ _ _._kilogram  Height.: _ _ _._cm | Continuous variable. Body mass index was calculated as weight (in kg) divided by the square of height (in meters). |
| **Control variables** |  |  |  |
| Age | Date of birth:_year_month_day | Birth date | Continuous variable |
| Marital status | What is your marital status? | 1=Never married 2=Married 3=Divorced 4=Widowed 5=Separated 9=Unknown | 1=Married 0=Not married (never married, divorced, widowed, or separated). Any response marked as "unknown" was treated as missing data |
| Place of residence | Urban/rural site: | 1=Urban site 2=Rural site | 1=Urban residence 0=Rural residence |
| Educational attainment | What is the highest level of education you have attained? | 0=None  1=Graduated from primary school  2=Lower middle school degree 3=Upper middle school degree 4=Technical or vocational degree  5=University or college degree 6=Master’s degree or higher 9=Unknown | We categorized participants' educational attainment into four groups: primary education or below (serving as the reference group), lower secondary education, upper secondary education, and tertiary education or above. Subsequently, the categories of lower secondary education, upper secondary education, and tertiary education or above were coded as dummy variables |
| Household income per capita (in thousands of Chinese yuan) | There are questions about nine potential sources of income in the questionnaires: business, subsidies, and other income | After calculating household income from each source, total household income was constructed as the sum from all nine sources. Household income per capita was calculated by dividing total household income by household size. To account for Household income per capita was adjusted for inflation, the household income per capita was adjusted using the 2015 Consumer Price Index | Continuous variable |
| Employment | Are you presently working? | 1=Yes 0=No | 1=Employed 0=Unemployed |
| Smoking | Have you ever smoked cigarettes (including hand-rolled or device-rolled)? | 0=Never smoked 1=Yes 9=Unknown | 1=Ever smoked 0=Never smoked. Any response marked as "unknown" was treated as missing data |
| Frequent alcohol drinking | In the year of survey, did you drink beer or any other alcoholic beverage? How often did you drink beer or any alcoholic beverage? | Drink alcoholic beverage or not: 0=No 1=Yes 9=Unknown  Frequency of alcohol drinking: 1=Almost every day 2=3-4 times a week 3=Once or twice a week 4=Once or twice a month 5=No more than once a month 9=Unknown | In accordance with the classifications used in prior research by Pan & Palmer (2018) ^a^, individuals who consume alcohol more than 3-4 times per week were categorized as frequent alcohol drinkers. |
| Loss to follow-up | Individuals who participated in previous survey waves but did not attend the 2015 wave and were not deceased were classified as lost to follow-up. | | Consistent with prior research ^b, c^, we constructed two time-invariant dummy variables to indicate loss to follow-up and death, respectively, to account for the potential selection bias |
| Death | Date of death:_year_month_day | Year of death |  |
| Province | Province: | 23=Heilongjiang 21=Liaoning 37=Shandong 41=Henan 32=Jiangsu 42=Hubei 43=Hunan 52=Guizhou 45=Guangxi 11=Beijing 31=Shanghai 55=Chongqing | Beijing was used as the reference group. The remaining eleven provincial-level regions were represented using dummy variables. |

^a^ Pan, T., & Palmer, M. (2018). Risk factors and non-communicable disease diagnosis in China. China Economic Review, 50, 72-84.

^b^ Chen, F., Yang, Y., & Liu, G. (2010). Social change and socioeconomic disparities in health over the life course in China: A cohort analysis. American Sociological Review, 75(1), 126-150.

^c^ Wu, J., Muennig, P. A., Keyes, K., & Wu, J. (2019). Generational differences in longitudinal blood pressure trajectories by geographic region during socioeconomic transitions in China. International Journal of Public Health, 64, 1375-1387.

**Table S2** Estimates from multilevel growth-curve models of body mass index (BMI), China Health and Nutrition Survey (1991-2015)

|  | Model 1 |
| --- | --- |
|  | Coefficient  (95% CI^a^) |
| **Fixed effects** |  |
| Intercept | 23.91 (23.61-24.22) |
| Male (Reference=Female) | -0.38 (-0.60--0.17) |
| Birth cohort | 0.05 (0.04-0.06) |
| Male*Birth cohort | 0.05 (0.03-0.06) |
| **Control variables** |  |
| Age | 0.12 (0.11-0.13) |
| Age squared | -0.00 (-0.00--0.00) |
| Male*Age | 0.00 (-0.00-0.01) |
| Male*Age squared | -0.00 (-0.00--0.00) |
| Birth cohort*Age | 0.00 (-0.00-0.00) |
| Birth cohort*Age squared | 0.00 (0.00-0.00) |
| Married (Reference=Not married) | 0.07 (-0.02-0.17) |
| Urban residence (Reference=Rural residence) | 0.10 (-0.00-0.21) |
| Educational attainment (Reference=Primary education or below) |  |
| Lower secondary education | 0.02 (-0.05-0.10) |
| Upper secondary education | 0.02 (-0.09-0.12) |
| Post-secondary education | -0.09 (-0.21-0.04) |
| Household income per capita (in thousands of Chinese yuan) | -0.00 (-0.00-0.00) |
| Employed (Reference=Not employed) | -0.06 (-0.12--0.01) |
| Ever smoking (Reference=Never smoking) | -0.19 (-0.26--0.12) |
| Frequent alcohol drinking (Reference=No frequent alcohol drinking) | 0.07 (-0.00-0.14) |
| Loss to follow-up | -0.03 (-0.13-0.08) |
| Death | -0.30 (-0.62-0.02) |
| Provincial fixed effect | Yes |
| **Random-effect variance components** |  |
| Individual-level: in intercept | 6.36 |
| Individual-level: in slope | 0.01 |
| Residual | 3.27 |
| AIC ^b^ | 229,515.19 |
| Number of respondents (Number of observations) | 14,375 (49,467) |

^a^ 95% CI, Confidence interval.

^b^ AIC, Akaike information criterion.

**Table S3** Estimates from multilevel growth-curve models of hypertension, China Health and Nutrition Survey (1991-2015)

|  | Model 1 | Model 2 | Model 3 |
| --- | --- | --- | --- |
|  | Coefficient  (S.E. ^a^) | Coefficient  (S.E. ^a^) | Coefficient  (S.E. ^a^) |
| **Fixed effects** |  |  |  |
| Intercept | -3.118 (0.180)*** | -2.955 (0.188)*** | -3.401 (0.183)*** |
| Male (Reference=Female) | 0.868 (0.072)*** | 0.547 (0.132)*** | 0.614 (0.127)*** |
| Birth cohort | 0.042 (0.005)*** | 0.028 (0.007)*** | 0.024 (0.006)*** |
| Male*Birth cohort |  | 0.025 (0.009)** | 0.014 (0.008) |
| Body mass index (kg/m^2^) |  |  | 1.107 (0.042)*** |
| **Control variables** |  |  |  |
| Age | 0.138 (0.009)*** | 0.132 (0.009)*** | 0.112 (0.009)*** |
| Age squared | 0.001 (0.000) | 0.001 (0.000) | 0.001 (0.000)** |
| Male*Age | -0.035 (0.005)*** | -0.027 (0.006)*** | -0.028 (0.005)*** |
| Male*Age squared | -0.000 (0.000) | -0.000 (0.000) | -0.000 (0.000) |
| Birth cohort*Age | 0.003 (0.001)*** | 0.003 (0.001)*** | 0.003 (0.001)*** |
| Birth cohort*Age squared | 0.000 (0.000) | 0.000 (0.000) | 0.000 (0.000) |
| Married (Reference=Not married) | 0.012 (0.083) | 0.013 (0.083) | -0.015 (0.081) |
| Urban residence (Reference=Rural residence) | -0.091 (0.056) | -0.091 (0.056) | -0.108 (0.053)* |
| Educational attainment (Reference=Primary education or below) |  |  |  |
| Lower secondary education | -0.076 (0.054) | -0.070 (0.054) | -0.082 (0.052) |
| Upper secondary education | -0.210 (0.070)** | -0.205 (0.070)** | -0.223 (0.068)** |
| Post-secondary education | -0.391 (0.085)*** | -0.388 (0.085)*** | -0.395 (0.082)*** |
| Household income per capita (in thousands of Chinese yuan) | -0.000 (0.001) | -0.000 (0.001) | -0.000 (0.001) |
| Employed (Reference=Not employed) | -0.107 (0.046)* | -0.108 (0.046)* | -0.093 (0.045)* |
| Ever smoking (Reference=Never smoking) | 0.023 (0.055) | 0.028 (0.055) | 0.075 (0.054) |
| Frequent alcohol drinking (Reference=No frequent alcohol drinking) | 0.258 (0.054)*** | 0.266 (0.054)*** | 0.274 (0.053)*** |
| Loss to follow-up | -0.007 (0.061) | -0.008 (0.061) | 0.003 (0.058) |
| Death | 0.570 (0.173)*** | 0.586 (0.172)*** | 0.631 (0.164)*** |
| Provincial fixed effect | Yes | Yes | Yes |
| AIC ^b^ | 604,625.729 | 604,625.138 | 604,366.256 |
| Number of respondents (Number of observations) | 14,375 (49,467) | 14,375 (49,467) | 14,375 (49,467) |

^a^ S.E., Standard error.

^b^ AIC, Akaike information criterion.

**Table S4** Estimates from multilevel growth-curve models of systolic/diastolic blood pressure (SBP/DBP), excluding people who had antihypertensive drugs, China Health and Nutrition Survey (1991-2015)

|  | SBP | | | DBP | | |
| --- | --- | --- | --- | --- | --- | --- |
|  | Model 1 | Model 2 | Model 3 | Model 1 | Model 2 | Model 3 |
|  | Coefficient  (S.E. ^a^) | Coefficient  (S.E. ^a^) | Coefficient  (S.E. ^a^) | Coefficient  (S.E. ^a^) | Coefficient  (S.E. ^a^) | Coefficient  (S.E. ^a^) |
| **Fixed effects** |  |  |  |  |  |  |
| Intercept | 110.962 (0.662)*** | 111.763 (0.682)*** | 111.440 (0.660)*** | 72.145 (0.458)*** | 72.652 (0.471)*** | 72.413 (0.455)*** |
| Male (Reference=Female) | 4.926 (0.248)*** | 3.165 (0.436)*** | 3.445 (0.419)*** | 3.784 (0.173)*** | 2.662 (0.301)*** | 2.859 (0.289)*** |
| Birth cohort | 0.377 (0.016)*** | 0.310 (0.021)*** | 0.262 (0.020)*** | 0.223 (0.011)*** | 0.180 (0.014)*** | 0.146 (0.014)*** |
| Male*Birth cohort |  | 0.139 (0.028)*** | 0.097 (0.027)*** |  | 0.089 (0.020)*** | 0.059 (0.019)** |
| Body mass index (kg/m^2^) |  |  | 0.861 (0.022)*** |  |  | 0.617 (0.015)*** |
| **Control variables** |  |  |  |  |  |  |
| Age | 0.727 (0.025)*** | 0.708 (0.025)*** | 0.612 (0.025)*** | 0.434 (0.017)*** | 0.421 (0.017)*** | 0.351 (0.017)*** |
| Age squared | 0.012 (0.001)*** | 0.012 (0.001)*** | 0.013 (0.001)*** | -0.004 (0.001)*** | -0.004 (0.001)*** | -0.003 (0.001)*** |
| Male*Age | -0.133 (0.014)*** | -0.093 (0.017)*** | -0.094 (0.016)*** | -0.030 (0.009)** | -0.003 (0.011) | -0.004 (0.011) |
| Male*Age squared | -0.004 (0.001)*** | -0.004 (0.001)*** | -0.003 (0.001)** | -0.003 (0.001)*** | -0.003 (0.001)*** | -0.002 (0.001)*** |
| Birth cohort*Age | 0.010 (0.002)*** | 0.010 (0.002)*** | 0.009 (0.002)*** | 0.003 (0.001)* | 0.003 (0.001)* | 0.003 (0.001)* |
| Birth cohort*Age squared | 0.000 (0.000)** | 0.000 (0.000)** | 0.000 (0.000)** | 0.000 (0.000)*** | 0.000 (0.000)*** | 0.000 (0.000)*** |
| Married (Reference=Not married) | -0.339 (0.257) | -0.346 (0.257) | -0.473 (0.252) | -0.305 (0.179) | -0.309 (0.179) | -0.403 (0.174)* |
| Urban residence (Reference=Rural residence) | -0.545 (0.195)** | -0.543 (0.195)** | -0.654 (0.186)*** | -0.244 (0.133) | -0.243 (0.133) | -0.316 (0.126)* |
| Educational attainment (Reference=Primary education or below) |  |  |  | -0.178 (0.129) | -0.156 (0.129) | -0.169 (0.124) |
| Lower secondary education | -0.555 (0.186)** | -0.521 (0.186)** | -0.551 (0.180)** | -0.178 (0.129) | -0.156 (0.129) | -0.169 (0.124) |
| Upper secondary education | -0.712 (0.242)** | -0.689 (0.242)** | -0.688 (0.234)** | -0.387 (0.167)* | -0.373 (0.167)* | -0.351 (0.161)* |
| Post-secondary education | -1.871 (0.296)*** | -1.851 (0.295)*** | -1.738 (0.285)*** | -0.958 (0.204)*** | -0.945 (0.204)*** | -0.837 (0.197)*** |
| Household income per capita (in thousands of Chinese yuan) | -0.003 (0.004) | -0.003 (0.004) | -0.003 (0.004) | 0.001 (0.003) | 0.001 (0.003) | 0.001 (0.002) |
| Employed (Reference=Not employed) | 0.142 (0.160) | 0.132 (0.160) | 0.218 (0.158) | 0.137 (0.114) | 0.130 (0.114) | 0.183 (0.112) |
| Ever smoking (Reference=Never smoking) | -0.033 (0.196) | 0.001 (0.196) | 0.231 (0.192) | -0.063 (0.138) | -0.040 (0.138) | 0.125 (0.135) |
| Frequent alcohol drinking (Reference=No frequent alcohol drinking) | 1.163 (0.197)*** | 1.206 (0.197)*** | 1.201 (0.194)*** | 0.723 (0.139)*** | 0.752 (0.140)*** | 0.757 (0.137)*** |
| Loss to follow-up | -0.128 (0.203) | -0.131 (0.203) | -0.132 (0.193) | 0.216 (0.138) | 0.214 (0.138) | 0.209 (0.131) |
| Death | 2.725 (0.597)*** | 2.810 (0.597)*** | 3.089 (0.569)*** | 0.923 (0.407)* | 0.976 (0.407)* | 1.197 (0.387)** |
| Provincial fixed effect | Yes | Yes | Yes | Yes | Yes | Yes |
| **Random-effect variance components** |  |  |  |  |  |  |
| Individual-level: in intercept | 53.064 | 52.871 | 44.583 | 23.680 | 23.616 | 19.469 |
| Individual-level: in slope | 0.110 | 0.110 | 0.102 | 0.023 | 0.023 | 0.020 |
| Residual | 118.359 | 118.319 | 117.810 | 62.686 | 62.669 | 62.402 |
| AIC ^b^ | 371,793.457 | 371,771.339 | 370,308.197 | 339,309.115 | 339,290.400 | 337,743.923 |
| Number of respondents (Number of observations) | 13,872 (46,973) | 13,872 (46,973) | 13,872 (46,973) | 13,872 (46,973) | 13,872 (46,973) | 13,872 (46,973) |

^a^ S.E., Standard error.

^b^ AIC, Akaike information criterion.

**Table S5** Estimates from multilevel growth-curve models of systolic/diastolic blood pressure (SBP/DBP), using the multiply imputed datasets, China Health and Nutrition Survey (1991-2015)

|  | SBP | | | DBP | | |
| --- | --- | --- | --- | --- | --- | --- |
|  | Model 1 | Model 2 | Model 3 | Model 1 | Model 2 | Model 3 |
|  | Coefficient  (S.E. ^a^) | Coefficient  (S.E. ^a^) | Coefficient  (S.E. ^a^) | Coefficient  (S.E. ^a^) | Coefficient  (S.E. ^a^) | Coefficient  (S.E. ^a^) |
| **Fixed effects** |  |  |  |  |  |  |
| Intercept | 117.791 (0.479)*** | 118.786 (0.511)*** | 117.797 (0.500)*** | 78.879 (0.340)*** | 79.544 (0.350)*** | 78.883 (0.339)*** |
| Male (Reference=Female) | 4.980 (0.229)*** | 2.987 (0.439)*** | 3.311 (0.410)*** | 3.990 (0.156)*** | 2.658 (0.325)*** | 2.879 (0.307)*** |
| Birth cohort | 0.371 (0.016)*** | 0.302 (0.023)*** | 0.245 (0.022)*** | 0.217 (0.011)*** | 0.171 (0.016)*** | 0.132 (0.016)*** |
| Male*Birth cohort |  | 0.142 (0.029)*** | 0.089 (0.028)** |  | 0.095 (0.021)*** | 0.058 (0.020)** |
| Body mass index (kg/m^2^) |  |  | 1.046 (0.021)*** |  |  | 0.727 (0.014)*** |
| **Control variables** |  |  |  |  |  |  |
| Age | 0.807 (0.027)*** | 0.785 (0.027)*** | 0.668 (0.026)*** | 0.471 (0.017)*** | 0.456 (0.017)*** | 0.375 (0.016)*** |
| Age squared | 0.013 (0.001)*** | 0.012 (0.001)*** | 0.014 (0.001)*** | -0.004 (0.001)*** | -0.004 (0.001)*** | -0.003 (0.001)*** |
| Male*Age | -0.140 (0.016)*** | -0.097 (0.019)*** | -0.102 (0.018)*** | -0.034 (0.011)** | -0.004 (0.013) | -0.008 (0.012) |
| Male*Age squared | -0.004 (0.001)*** | -0.004 (0.001)*** | -0.002 (0.001)* | -0.004 (0.001)*** | -0.003 (0.001)*** | -0.002 (0.001)*** |
| Birth cohort*Age | 0.008 (0.002)*** | 0.008 (0.002)*** | 0.007 (0.002)** | 0.001 (0.001) | 0.001 (0.001) | 0.001 (0.001) |
| Birth cohort*Age squared | 0.000 (0.000)* | 0.000 (0.000)* | 0.000 (0.000) | 0.000 (0.000)*** | 0.000 (0.000)*** | 0.000 (0.000)*** |
| Married (Reference=Not married) | -0.136 (0.293) | -0.198 (0.291) | -0.422 (0.286) | -0.034 (0.011)** | -0.004 (0.013) | -0.008 (0.012) |
| Urban residence (Reference=Rural residence) | -0.664 (0.176)*** | -0.675 (0.176)*** | -0.820 (0.168)*** | -0.462 (0.120)*** | -0.469 (0.120)*** | -0.558 (0.113)*** |
| Educational attainment (Reference=Primary education or below) |  |  |  |  |  |  |
| Lower secondary education | -0.410 (0.199)* | -0.374 (0.198) | -0.383 (0.196) | -0.127 (0.144) | -0.103 (0.144) | -0.107 (0.144) |
| Upper secondary education | -0.535 (0.232)* | -0.508 (0.233)* | -0.414 (0.220) | -0.477 (0.162)*** | -0.459 (0.162)** | -0.384 (0.156)* |
| Post-secondary education | -2.192 (0.286)*** | -2.714 (0.285)*** | -1.996 (0.274)*** | -1.213 (0.196)*** | -1.201 (0.196)*** | -1.057 (0.186)*** |
| Household income per capita (in thousands of Chinese yuan) | -0.006 (0.004) | -0.006 (0.004) | -0.007 (0.004)* | -0.000 (0.002) | -0.000 (0.002) | -0.001 (0.002) |
| Employed (Reference=Not employed) | -0.100 (0.162) | -0.113 (0.162) | 0.018 (0.161) | -0.052 (0.109) | -0.062 (0.109) | 0.027 (0.109) |
| Ever smoking (Reference=Never smoking) | -0.021 (0.200) | 0.020 (0.200) | 0.338 (0.193) | -0.126 (0.137) | -0.098 (0.137) | 0.127 (0.135) |
| Frequent alcohol drinking (Reference=No frequent alcohol drinking) | 1.250 (0.215)*** | 1.308 (0.215)*** | 1.291 (0.204)*** | 0.694 (0.151)*** | 0.733 (0.151)*** | 0.726 (0.145)*** |
| Loss to follow-up | -0.086 (0.206) | -0.093 (0.206) | -0.073 (0.196) | 0.365 (0.126)** | 0.359 (0.126)** | 0.364 (0.120)** |
| Death | 2.860 (0.624)*** | 2.939 (0.624)*** | 3.374 (0.592)*** | 1.112 (0.413)** | 1.163 (0.413)** | 1.472 (0.391)*** |
| Provincial fixed effect | Yes | Yes | Yes | Yes | Yes | Yes |
| Number of respondents (Number of observations) | 16,318 (70,318) | 16,318 (70,318) | 16,318 (70,318) | 16,318 (70,318) | 16,318 (70,318) | 16,318 (70,318) |

^a^ S.E., Standard error.
